# Associations of PON1 and Genetic Ancestry with Obesity in Early Childhood

**DOI:** 10.1371/journal.pone.0062565

**Published:** 2013-05-03

**Authors:** Karen Huen, Kim Harley, Kenneth Beckman, Brenda Eskenazi, Nina Holland

**Affiliations:** 1 Center for Environmental Research and Children's Health, School of Public Health, University of California, Berkeley, Berkeley, California, United States of America; 2 Biomedical Genomics Center, Institute for Health Informatics, University of Minnesota, Minneapolis, Minnesota, United States of America; Harvard Medical School, United States of America

## Abstract

Obesity in children has become an epidemic in the U.S. and is particularly prominent in minority populations such as Mexican-Americans. In addition to physical activity and diet, genetics also plays a role in obesity etiology. A few studies in adults and adolescents suggest a link between obesity and paraoxonase 1 (PON1), a multifunctional enzyme that can metabolize organophosphate pesticides and also has antioxidant properties. We determined *PON1_192_* genotype and arylesterase levels (ARYase, measure of PON1 enzyme quantity), to characterize the relationship between PON1 and obesity in young Mexican-American children (n = 373) living in an agricultural community in California. Since *PON1* polymorphisms and obesity both vary between ethnic groups, we estimated proportional genetic ancestry using 106 ancestral informative markers (AIMs). Among children, *PON1_192_* allele frequencies were 0.5 for both alleles, and the prevalence of obesity was high (15% and 33% at ages two and five, respectively). The average proportion of European, African, and Native American ancestry was 0.40, 0.09, and 0.51, yet there was wide inter-individual variation. We found a significantly higher odds of obesity (9.3 and 2.5- fold) in *PON1_192QQ_* children compared to *PON1_192RR_* children at ages two and five, respectively. Similar relationships were seen with BMI Z-scores at age two and waist circumference at age five. After adjusting for genetic ancestry in models of PON1 and BMI Z-score, effect estimates for *PON1_192_* genotype changed 15% and 9% among two and five year old children, respectively, providing evidence of genetic confounding by population stratification. However even after adjustment for genetic ancestry, the trend of increased BMI Z-scores with increased number of *PON1_192_* Q alleles remained. Our findings suggest that PON1 may play a role in obesity independent of genetic ancestry and that studies of PON1 and health outcomes, especially in admixed populations, should account for differences due to population stratification.

## Introduction

Childhood obesity rates have risen rapidly from 6.5 to 20% in the last 30 years[1], [2] and are particularly high among Latinos [2], [3], [4]. Studies have demonstrated that obesity early in life can be a risk factor for later chronic diseases such as cardiovascular disease and diabetes [5], making it a significant public health concern. Although overnutrition and sedentary behaviors are widely accepted as major contributors to the development of obesity in children, growing evidence suggests obesity is a complex condition that is also influenced by genes[6], [7] and the environment[8], [9], [10], [11].

Although genome-wide association studies (GWAS) of obesity have been performed in children[12], [13], only a few candidate genes have been identified and they explain only a small portion of the heritability of obesity[14]. One of the limitations of GWAS is the sheer number of multiple tests required when hundreds of thousands to over a million genetic variants are analyzed. Since obesity is a complex trait, the reduced power of adjusting for multiple testing can mask associations with some polymorphisms, such as paraoxonase 1 (PON1), which may also contribute to obesity heritability with small but significant effects[15].

PON1 is a high density lipoprotein (HDL)-associated enzyme that detoxifies the oxon derivatives of some organophosphate (OP) pesticides. It is also involved in prevention of lipid peroxidation and has been associated with diseases characterized by high oxidative stress, such as cardiovascular disease and diabetes[16], [17], [18]. Obesity, in particular, is considered a condition of chronic oxidative stress[19]. Data on the relationship between PON1 and obesity, especially in children, are limited[20]. In adults, a few small studies have reported a trend of lower PON1 activity in obese versus normal weight individuals[21], [22], [23]. In the only study of children, 12-year-olds (N = 110) with BMIs >95^th^ percentile had significantly lower PON1 levels in comparison to normal weight controls[17].

Data on the relationship between obesity and PON1 genotypes has been limited and inconsistent. Among Portuguese women, the odds of obesity were higher among women with the *PON1_192_* QR or RR genotypes but no association was seen with the *PON1_L55M_* genotype [24]. In contrast, in a Mexican population [25], the *PON1_55_* LL genotype but not the *PON1_192_* QR or RR genotype was associated with obesity. Obesity is also an important risk factor for cardiovascular disease. The *PON1_192_* QQ genotype has also been associated with increased risk of major cardiovascular events in a cohort of white and African-American adults[16], however among school-aged children prenatally exposed to pesticides, the RR genotype was associated with adverse cardiovascular risk profiles[26].

Despite pronounced differences in PON1 allele frequencies and obesity prevalence between ethnic groups, no studies of PON1 genetics and obesity have adjusted for potential genetic confounding. For instance, the frequency of the Q allele for the *PON1_192_* SNP is 0.73 for Caucasians[27], 0.37 for African-Americans[27] and 0.48 for Mexicans[28], [29]. Furthermore, it is well established that prevalence of obesity differs among different ethnic and racial groups even after controlling for socioeconomic status[30]. In genetic association studies of admixed populations, heterogeneity of genetic background can lead to spurious associations if ancestry is related to both a candidate gene and the disease outcome of interest; this is also referred to as genetic confounding due to population stratification[31]. Structured association methods enable us to adjust for potential genetic confounding. Briefly, proportions of genetic admixture are estimated using ancestral informative markers (AIMs), unlinked genetic markers whose frequencies differ substantially between ancestral groups. Estimated proportion can then be included in statistical models as covariates. To our knowledge, only one study has attempted to adjust for population stratification in *PON1* genetic association studies and this was in relation to PON1 enzymatic activities[32]. Furthermore since studies suggest genetic admixture may influence obesity parameters[33], [34], it is of critical importance to adjust for genetic ancestry as a potential confounder in studies of PON1 and obesity in admixed populations.

Previously, we explored the association of PON1 genotypes and activities with birth outcomes and fetal growth in newborns from the Center for Health Assessment of Mothers and Children of Salinas(CHAMACOS) study and found that lower PON1 activity was associated with shorter gestational age and smaller head circumference in newborns[35]. Other studies have demonstrated that PON1 status, which includes measures of both *PON1_192_* genotype and protein levels, may be a more comprehensive descriptor of PON1 molecular phenotype and a more accurate predictor of disease [36], [37], [38]. In this paper, we extend our studies of the CHAMACOS birth cohort to examine the association of PON1 status (modeled as *PON1_192_* genotype and arylesterase activity) with outcomes of growth and obesity in young children at ages two and five years. We also determine the relationship between genetic ancestry and childhood obesity in these children and additionally whether it confounds the effect of PON1 on obesity.

## Materials and Methods

### Ethics Statement

Study protocols were approved by the University of California, Berkeley Committee for Protection of Human Subjects. Written informed consent for the participation of mothers and their children in the study was obtained from all mothers. Consent was obtained at the time of enrollment by CHAMACOS staff, who provided a thorough explanation of the study and answered all questions. Follow-up consents were obtained at the time of delivery and at each postpartum contact point. Because of the low literacy level of the population, all consent forms were read aloud by the study interviewer.

### Study population

The CHAMACOS study is a longitudinal birth cohort study of the effects of pesticides and other environmental exposures on children's neurodevelopment, growth, and respiratory disease [39]. Study participants live and work in the Salinas Valley in Monterey County, CA, an agricultural region. Pregnant women who were receiving prenatal care in one of six community clinics were enrolled in the study between 1999 and 2000. Women eligible to participate in the study were at least 18 years of age, spoke English or Spanish, qualified for Medicaid, and were less than 20 weeks gestation. Participants were primarily Latina and most were born in Mexico. Six hundred and one pregnant women were enrolled and 527 delivered liveborn singleton newborns. Follow-up visits occurred when the children were 6 months, and 1, 2, 3½, and 5 years old. For this analysis, we include those who were followed up for anthropometric measurements at age 2 (n = 386) and 5 (n = 331), and had blood samples collected at both time points. We further limit the study to those children who were genotyped for *PON1_192_* and AIMs (N = 373 children total, 360 and 311 had anthropometric measurements at 2 years and 5 years of age, respectively). A smaller portion of children also had adequate heparinized plasma volumes for analysis of ARYase activity at those ages (n = 243 and 215 for two and five year olds, respectively). There were no significant differences in demographic or anthropometric characteristics between the subset of children included in these analyses and all the children in the CHAMACOS cohort.

### Anthropometric measurements

Maternal pre-pregnancy BMI was calculated using self-reported pre-pregnancy weight when interviewed at enrollment (mean ± SD, 14.0 ± 5.0 weeks gestation), and measured height. Children's height and weight were measured at each visit and waist circumference was measured at age 5 [40], [41], [42]. Height without shoes was measured using a stadiometer. Waist circumference was calculated with a measuring tape placed around the abdomen at the iliac crest. Height and waist measurements were performed in triplicate and then averaged. Child weight was determined using a calibrated electronic scale (Tanita Mother-Baby Scale Model 1582, Tanita Corp). To ensure reliability, periodic refresher trainings were performed and measurements were compared among interviewers. BMI, calculated as weight divided by height^2^ (kg/m^2^) was compared to CDC reference data[43] to generate BMI Z-scores standardized by sex and age. Children with a BMI at or above the 95^th^ percentile of the 2000 CDC sex-specific BMI-for-age growth charts,[44] were considered obese.

### Blood collection and processing

Blood specimens were collected from the umbilical cords of CHAMACOS children after delivery for the determination of PON1 and AIMs genotypes. Genotypes were determined using DNA isolated from blood clots as described previously. [28]. Heparinized whole blood was collected in BD vacutainers® (Becton, Dickinson and Company, Franklin Lakes, NJ), centrifuged, divided into plasma, buffy coats and red blood cells, and stored at -80°C at the School of Public Health Biorepository, University of California, Berkeley. Measurement of ARYase activity was performed in heparinized plasma collected from children at two (mean±SD, 2.01 ± 0.09 yr) and five (mean±SD, 5.10 ± 0.22 yr) years of age. Stringent conditions in accordance with the Best Practices for Biorepositories were followed [45].

### Determination of PON1 ARYase activity levels

ARYase activity was measured by determining the rate of phenyl acetate hydrolysis in plasma samples using spectrophotometric methods as described previously [46]. ELISA and Western blot based methods utilizing PON1 antibodies confirm a high correlation (r>0.85) between measured PON1 quantity and ARYase activity [47], [48]. Therefore, the ARYase assay is considered a measure of PON1 enzyme quantity. All assays were performed in triplicate. Quality assurance, described in more detail in Huen et al. [49], included assessment of repeat samples (separate aliquots of the same sample run on different days), internal controls (aliquots of the same sample run on all assay plates), and concurrent analyses of specimens from different collections (samples from different time points run on the same plates). Repeated analysis of 3% of samples showed a high degree of concordance. The average coefficient of variation (CV) for repeated samples was 8.5% and the correlation coefficient between repeated runs was 0.94. Inter-assay variability, as measured by the average CV for internal controls samples was 8.7%.

### AIMs selection

One hundred and six SNPs were used as AIMs for determination of genetic ancestry among CHAMACOS participants, as it has previously been established that a panel of this size is sufficient to accurately estimate genetic ancestry in Latino populations[50]. We chose to use the same panel of AIMs as has been reported in other studies of Latino populations [51], [52], [53]. Briefly, SNPs were selected if the delta (difference between allele frequencies) between pairs of ancestral populations (Western African, European, or Native American) were greater than 0.5, allowing for maximization of information gleaned from each SNP. Selected AIMs were widely distributed across the genome among all 22 autosomes with an average distance of 2.4 × 10^7^ bp between each marker.

### Determination of genotypes (*PON1_192_* and AIMs)

Previously, we sequenced the *PON1* gene in CHAMACOS subjects and determined the functional significance of over 44 PON1 SNPs[54]. PON1 molecular phenotype is strongly influenced both by enzyme quantity and catalytic efficiency. We found that the *PON1_192_* genotype was the strongest predictor of catalytic efficiency, as measured by substrate-specific paraoxonase (POase) activity, and other SNPs including the coding SNP *PON1_55_* only explained a moderate amount of additional phenotypic variation. The promoter SNP *PON1_−108_* is known to significantly affect PON1 protein levels, as measured by arylesterase activity, however even in combination with other promoter polymorphisms, it only explains 26% of the variation of PON1 quantity in CHAMACOS children[55]. Therefore, we chose to focus our study on the *PON1_192_* SNP and arylesterase activity as a comprehensive measure of PON1 status. PON1 status, which accounts for both PON1 catalytic efficiency and enzyme quantity, can be more informative than looking at PON1 genotype alone in epidemiologic studies[46].

The coding polymorphism, *PON1_192_* was genotyped using the Taqman real-time polymerase chain reaction (PCR) method. Briefly, primers for the nucleotide sequence flanking the SNP, and probes specific for the SNP were custom-designed by Applied Biosystems, Inc. (Foster City, CA). AIMs were genotyped using the multiplex platform iPLEX (Sequenom, San Diego, CA) as described previously[56]. Four multiplex assays were used to determine genotypes for 106 AIMs (all SNPs). The main steps involved multiplex PCR, single-base primer extension, and finally mass spectrometry to determine the genotype.

Quality assurance procedures for genotyping included assessment of randomly distributed blank samples in each plate and duplicates of randomly selected samples with independently isolated DNA from the same subjects. Repeated analysis (4% of samples) in several runs showed a high degree (>99%) of concordance. All discrepancies were resolved with additional genotyping.

#### Estimation of Genetic Ancestry

We used STRUCTURE 2.3.3 software [57], [58], [59], which applies a Bayesian approach, to estimate the proportion of genetic ancestry for each CHAMACOS participant. An admixture model with independent allele frequencies was performed with a burn-in period of 50,000, followed by 50,000 iterations after burn-in, and K = 3. This analysis generated proportional ancestry estimates for each of the three ancestral populations (African, European, and Indigenous American) based on the known frequencies in 35 West Africans, 40 Europeans, and 28 Indigenous Americans.

### Statistical Analysis

We used a chi-squared goodness of fit test to assess whether allele frequencies for each polymorphism (106 AIMs and *PON1_192_*) deviate from Hardy-Weinberg equilibrium.

We used regression models to determine the associations between the *PON1_192_* genotype and obesity parameters at ages 2 and 5. *PON1_192_* genotype was expressed in two ways: categorically as QQ, QR, or RR and ordinally as 0, 1, or 2 for the number of Q alleles. We incorporated a measure of PON1 status by including variables for both ARYase and *PON1_192_* genotype within the same statistical models and considered their interaction.

Linear regression models were performed for the continuous outcomes BMI Z-score (both ages) and waist circumference (only age five) and logistic regression models were employed for obesity status (coded at above and below the 95^th^ percentile). In this study, we chose to focus on obese children, although trends for overweight children were similar (data not shown). For linear and logistic regression models including PON1 status, we retained the interaction term for *PON1_192_* × ARYase in the model if the F-test comparing the full model with the interaction term to the nested model with no interaction term was statistically significant (p<0.20).

To determine which covariates should be included in the models, we tested for the association of demographic (e.g. maternal country of birth, maternal age during pregnancy, maternal education), diet (e.g. soda, fruit, and vegetable consumption), and physical activity (time spent outside, hours of television watched) parameters with PON1 and found no significant associations except for maternal BMI, child birthweight, and genetic ancestry. Only a few factors were significantly associated with obesity parameters at age two (maternal BMI, birthweight, child soft drink consumption[40], and genetic ancestry) and five (maternal BMI, birthweight). Although birthweight was associated with both PON1 genotype and obesity, it was likely an intermediate variable such that PON1 genotype influences birthweight[35] and then birthweight in turn influences obesity. Therefore, we chose not to include it in our models as it has been shown that controlling for intermediary variables leads to overadjustment and can bias results towards the null[60]. Furthermore, maternal BMI was not included in our models because adjusting for maternal obesity may overcontrol for the relationship between genetic ancestry and child obesity. To examine the possibility of confounding by population heterogeneity, we added genetic ancestry estimates as continuous variables to the models looking at effects of PON1 on obesity parameters at ages 2 and 5. Since the sum of the admixture proportions for all three ancestral groups (European, Native American, and African) is equal to one, the three variables are collinear. Therefore, we included only two of the admixture proportions (European and African) in the models. Beta estimates or odds ratio for PON1 genotype that differed by more than 10% after adjusting for admixture proportions were considered as evidence of genetic confounding. Since we performed numerous tests over three measurements of obesity (only two at age two) at two ages (5 tests total per model), we used Bonferonni correction in which a p-value less than 0.01(α = 0.05/5) was considered to be significant after adjusting for multiple testing.

As a secondary analysis, we also examined the direct relationship between genetic ancestry and obesity parameters at ages two and five. We constructed linear and logistic regression models where the outcome was the obesity parameter of interest (e.g. BMI Z score, obesity status, or waist circumference) and the independent variable was the proportion of European, Native American, or African ancestry. Separate models were run for each ancestral group. European ancestry and Native American ancestry were coded continuously as percent ancestry. Since the distribution of African ancestry was right-skewed, it was log transformed to normalize the distribution. Again we used Bonferonni adjustment for n = 15 tests and considered a p-value less than 0.003 to be statistically significant. All analyses were performed in Stata 11.2 (College Station, TX).

## Results

### Participant Characteristics

Maternal and child characteristics and information on child diet and activity are presented in **Table 1**. At the time of birth, mothers were primarily young (mean ± SD, 25.6 ±5.3 years), low-income, and Mexican-born. Furthermore, the majority were either overweight or obese before their pregnancy. There was a relatively even distribution of CHAMACOS boys and girls included in this analysis.

**Table 1:** Study Population Demographics and Child Diet and Physical Activity Parameters.

	No.	%
**Maternal Characteristics**		
Pre-Pregnancy BMI		
Underweight (<18.5)	1	0.3
Normal (18.5 – 24.9)	132	35.4
Overweight (25 – 29.9)	149	39.9
Obese (>30)	91	24.4
Maternal Age at Delivery (years)		
18–24	164	44.0
25–29	121	32.4
30–34	59	15.8
35–45	29	7.8
Years Lived in US at Time of Delivery		
≤1	86	23.1
2–5	99	26.5
6–10	96	25.7
11+	56	15.0
Entire life	36	9.7
Maternal Education		
≤ 6th grade	166	44.5
7–12th grade	137	36.7
≥High School Graduate	70	18.8
**Child Characteristics**		
Sex		
Boy	179	48
Girl	194	52
Diet and Physical Activity^a^		
Soda consumption (nondiet) at age 2		
<1 per week	160	44.6
1–6 per week	154	42.9
1+ per day	45	12.5
Soda Consumption (nondiet) at age 5		
<1 per week	119	38.3
1–6 per week	165	53.1
1+ per day	27	8.7
Average daily TV time at age 5		
<1 hr/day	73	23.5
1–2 hrs/day	106	34.1
2+ hrs/day	132	42.4
Average daily outdoor play at age 5		
<1 hr/day	44	14.2
1–2 hrs/day	157	50.8
3–4 hrs/day	82	26.5
5+ hrs/day	26	8.4

a Total number of observations vary due to missing data.

### Obesity parameters among CHAMACOS children

At age two, child BMI Z-scores ranged from −5.1 to 4.4 with a mean ±SD of 0.47±1.2. In five year olds, the mean±SD BMI Z-score was 1.2±1.1 and ranged from −2.5 to 4.2. Waist circumference among five year olds ranged from 46.3 to 92.7 cm with a mean±SD of 58.6±7.7. Over 15% of two-year olds and 33% of five-year olds were obese (BMI Z-score ≥ 95^th^ percentile). The prevalence of obesity was higher in CHAMACOS children at both ages two and five than Mexican-American participants of the NHANES study (ages two through five)[1].

### *PON1_192_* Genotype and ARYase Activity

Among CHAMACOS children, the allele frequencies were 0.51 and 0.49 for the *PON1_192_* Q and R alleles, respectively. We observed broad variability of ARYase activity among CHAMACOS children ranging from 8.5–151.2 U/mL in two year olds and 22.4 to 157.9 U/mL in five year olds[61]. We found no significant differences in mean ARYase activities among two and five year old children.

### AIMs and Genetic Ancestry

**Figure 1:**
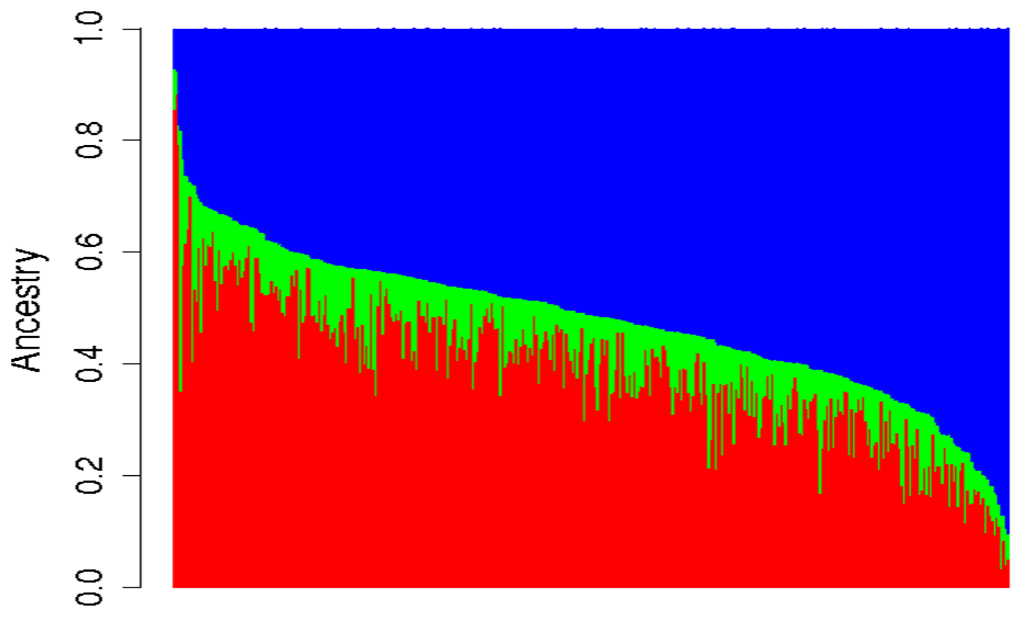
Bar plot of genetic ancestry estimates from STRUCTURE software (percent European, African, and Native American) in CHAMACOS children (n = 375). The red, green, and blue lines represent proportional European, African, and Native American ancestry, respectively.

Minor allele frequencies for AIMs ranged from 0.02 to 0.5 and the average was 0.3. Twenty-three AIMs significantly deviated from Hardy-Weinberg equilibrium. Among all CHAMACOS children (n =  373), the average proportion of European, African, and Native American ancestry was 0.39 (range  = 0.03–0.8), 0.09 (range = 0.02–0.47), and 0.52 (range = 07–0.91), respectively. Similar proportions were also observed in CHAMACOS mothers. **Figure 1** shows a bar plot representing the estimated proportions of genetic ancestry for all three ancestral groups for each CHAMACOS child. The figure shows great variability between individuals. Participants on the left side of the plot have high European ancestry, while those on the right side of the plot tend to have much higher proportions of Native American ancestry. On average, the proportion of African ancestry among Mexican-American CHAMACOS participants was quite low although the range was broad.

### PON1, BMI, and Obesity

We observed a significant association of child *PON1_192_* genotype and PON1 status with both BMI Z-score and obesity status at age two. When we coded *PON1_192_* genotype categorically, we observed a consistent detrimental effect of the Q allele. For example, compared to RR children, the odds of obesity (95% confidence interval) were 5.3 (1.6–18.0) and 9.3 (2.7–32.1) fold higher in QR and QQ children, respectively (p for trend = 0.01). As can be seen in the PON1 status model (model 2 including both ARYase and *PON1_192_* genotype in the same model) shown in **Table 2**, there was an independent association of *PON1_192_* and ARYase on BMI Z-score for two year olds: a standard deviation increase in ARYase activity was associated with a 0.2 unit increase in BMI Z-score (p = 0.01) while after adjusting for ARYase activity each Q allele was marginally associated with a 0.2 unit increase in BMI Z-score (p = 0.06) in two year old children. Similar trends were seen with obesity status in which increased ARYase and the number of Q alleles were associated with increased odds of obesity in children (p = 0.02 for both), but these were not considered statistically significant after adjustment for multiple testing. We observed no significant interaction between ARYase and *PON1_192_* at age two. Therefore an interaction term was not included in the model.

**Table 2:** Associations of PON1 with Obesity Parameters at Ages 2 and 5.

	Age 2				Age 5			
	N	β or OR(95% CI)	p-value^c^	r^2^	N	β or OR(95% CI)	p-value	r^2^
BMI Z score								
Model 1								
*PON1_192_* a	360	0.25(0.07,0.42)	0.01	0.02	311	0.15(−0.03,0.33)	0.10	0.01
Model 2								
ARYase^b^	243	0.19(0.04,0.33)	0.01	0.05	215	0.04(−0.11,0.19)	0.59	0.01
*PON1_192_*		0.20(−0.01,0.40)	0.06			0.11(−0.10,0.32)	0.32	
Waist Circumference								
Model 1								
*PON1_192_*					311	1.30(0.09,2.51)	0.04	0.01
Model 2								
ARYase					215	1.89(−0.04,3.81)	0.05	0.03
*PON1_192_*						4.61(−0.68,9.90)	0.09	
ARYase × *PON1_192_*						−0.98(−2.33,0.36)	0.15	
Obesity Status								
Model 1								
*PON1_192_*	360	2.41(1.54,3.76)	0.0001		311	1.55(1.10,2.18)	0.01	
Model 2								
ARYase	243	1.58(1.07,2.32)	0.02		215	1.73(0.87,3.46)	0.12	
*PON1_192_*		1.87(1.09,3.22)	0.02			5.05(0.79,32.33)	0.09	
ARYase × *PON1_192_*						0.70(0.44,1.11)	0.13	

a PON1_192_ coded as number of Q alleles, 0, 1 or 2.

b Children were considered obese if their BMI was at or above 95^th^ percentile of the 2000 CDC sex-specific BMI-for-age growth charts.

c Using a Bonferonni correction for multiple testing (5 tests per model), we considered p-values less than 0.01 to be statistically significant.

Model 1 examines the association of *PON1_192_* only with obesity parameters. Model 2 examines the association of PON1 status with obesity parameters by including both ARYase and *PON1_192_*.

We observed similar trends between child *PON1_192_* genotype and waist circumference, obesity status, and BMI Z-score in five year olds children. With an increasing number of *PON1_192_* Q alleles, we observed increased waist circumference (β(95%CI):1.3(0.09–2.51) and also increased odds (1.55 fold) of obesity in 5 year olds (**Table 2**). For example, compared to RR children the odds of obesity was 2.0 and 2.5 in QR and QQ children, respectively (p for trend = 0.01). After adjusting for multiple hypothesis testing however only the relationship with obesity status remained statistically significant. The PON1 status models for five year olds indicated a statistically significant interaction between ARYase and *PON1_192_* genotype for waist circumference and obesity status (p = 0.15 and 0.13, respectively, Table 2). To interpret the significant interaction terms in the waist circumference and obesity status, we created models of the association of ARYase on these outcomes, stratifying by *PON1_192_* genotype. Although cell numbers were relatively small for these models, they indicated a protective but non-significant trend of smaller waist circumference β(95%CI):−0.45(−2.15,1.24) and decreased odds of obesity OR(95%CI):0.81(0.50–1.30) for a SD increase in ARYase activity in QQ children. In contrast, among QR and RR children, we observed the opposite trend of larger waist circumference and increased odds of obesity with an SD increase in ARYase activity. However, this relationship was only significant for increased waist circumference among QR children β(95%CI):1.73(0.15,3.30).

### PON1, Genetic Ancestry, and Obesity

**Figure 2:**
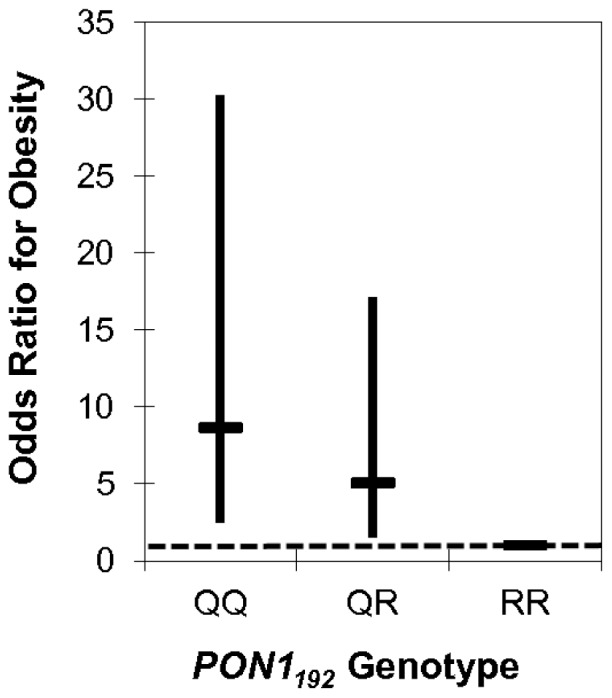
Odds of obesity by *PON1_192_* genotype in 2 year old CHAMACOS children. Compared to RR children, the OR(95%CI) for QQ and QR two year olds was 8.63(2.46–30.24) and 5.06(1.49–17.14), respectively (n = 360), after adjusting for genetic ancestry. The genotypic frequencies for these children were: QQ-25% QR-51% RR- 24%. The dashed line indicates an OR of 1.

To determine whether confounding by population stratification significantly affects the relationship between PON1 and obesity we looked at their associations while adjusting for proportional ancestry. The coefficient for *PON1_192_* genotype changed 15% and 9%, respectively at ages two and five in the models for BMI Z-score, providing some suggestive evidence of genetic confounding by population stratification (**Table 3**). Coefficients in the models for waist circumference and obesity status did not change substantially (2–3%). For example, **Figures 2** **and** **3** show that the significant association of odds of obesity with *PON1* genotype remains at both ages even after adjusting for genetic ancestry.

**Figure 3:**
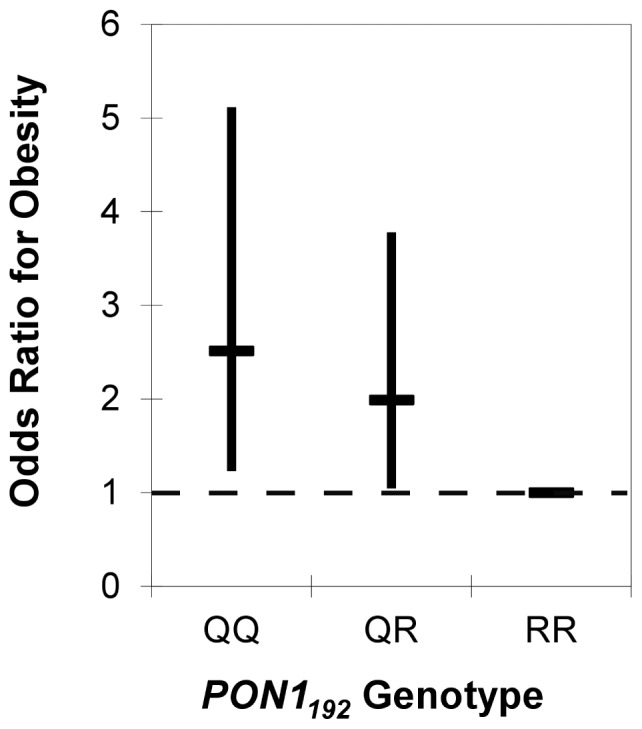
Odds of obesity by *PON1_192_* genotype in 5 year old CHAMACOS children. Compared to RR children, the OR(95%CI) for QQ and QR five year olds was 2.47(1.20–5.10) and 1.97(1.03–3.74), respectively(n = 311), after adjusting for genetic ancestry. The genotypic frequencies for these children were: QQ-25% QR-51% RR-24%. The dashed line indicates an OR of 1.

**Table 3:** Associations of Child PON1 and Genetic Ancestry with Obesity Parameters at Ages 2 and 5.

	Age 2				Age 5			
	N	β or OR(95% CI)	p-value^d^	r^2^	N	β or OR(95% CI)	p-value	r^2^
BMI Z-score								
ARYase	243	0.19(0.04,0.33)	0.011	<0.005	215	0.05(−0.10,0.21)	0.517	0.01
*PON1_192_* a		0.17(−0.03,0.38)	0.102			0.10(−0.11,0.32)	0.345	
% European Ancestry		0.13(−0.96,1.21)	0.815			−0.17(−1.36,1.02)	0.781	
% African Ancestry^b^		0.16(−0.10,0.41)	0.223			0.18(−0.11,0.47)	0.225	
Waist Circumference								
ARYase					215	2.00(0.05,3.94)	0.044	0.04
*PON1_192_*						4.75(−0.57,10.08)	0.080	
ARYase × *PON1_192_*						−1.03(−2.38,0.32)	0.135	
% European Ancestry						−1.46(−8.96,6.05)	0.702	
% African Ancestry						1.03(−0.79,2.85)	0.266	
Obesity Status^c^								
ARYase	243	1.58(1.07,2.34)	0.021		215	1.75(0.88,3.52)	0.113	
*PON1_192_*		1.91(1.09,3.34)	0.024			5.16(0.80,33.11)	0.084	
ARYase × *PON1_192_*						0.69(0.44,1.11)	0.125	
% European Ancestry		0.18(0.01,3.32)	0.250			0.71(0.07,7.24)	0.770	
% African Ancestry		1.57(0.78,3.14)	0.203			1.10(0.62,1.96)	0.735	

a PON1_192_ coded as number of Q alleles, 0, 1 or 2.

b Proportional African ancestry was log transformed to normalize the distribution.

c Children were considered obese if their BMI was at or above 95^th^ percentile of the 2000 CDC sex-specific BMI-for-age growth charts.

d Using a Bonferonni correction for multiple testing (5 tests per model), we considered p-values less than 0.01 to be statistically significant.

### Genetic Ancestry and Obesity

At age two, proportional European and Native American ancestry did not appear to be significantly associated with obesity in CHAMACOS children (**Table 4**). However, we did observed a trend of increased African ancestry (log transformed) with higher BMI Z-scores (β(95%CI):0.28(0.06–0.50)) and an increased odds of obesity (p = 0.02). These relationships were not statistically significant after adjusting for multiple testing. We also observed a similar pattern in five year old children where children with higher African ancestry had larger waist circumferences and increased odds of obesity, but these trends were not statistically significant (**Table 4**).

**Table 4:** Associations of Proportional Genetic Ancestry with Obesity Parameters at Ages 2 and 5.

	Age 2				Age 5			
	N	β or OR(95% CI)	p-value^c^	r^2^	N	β or OR(95% CI)	p-value	r^2^
BMI Z-score								
%European Ancestry	362	0.02(−0.92,0.95)	0.970	<0.0005	312	−0.34(−1.32,0.65)	0.502	0.001
% Native American Ancestry	362	−0.43(−1.36,0.50)	0.361	0.002	312	0.09(−0.89,1.08)	0.850	<0.0005
% African Ancestry^a^	362	0.28(0.06,0.50)	0.012	0.017	312	0.14(−0.08,0.37)	0.212	0.005
Waist Circumference								
%European Ancestry					313	−3.00(−9.72,3.72)	0.380	0.002
% Native American Ancestry					313	1.85(−4.87,8.57)	0.589	<0.0005
% African Ancestry					313	0.75(−0.78,2.28)	0.335	0.003
Obesity Status^b^								
%European Ancestry	362	0.62(0.07,5.36)	0.663		312	0.83(0.13,5.29)	0.842	
% Native American Ancestry	362	0.65(0.08,5.60)	0.699		312	0.86(0.13,5.49)	0.869	
% African Ancestry	362	1.91(1.11,3.29)	0.020		312	1.28(0.83,1.96)	0.267	

a Proportional African ancestry was log transformed to normalize the distribution.

b Children were considered obese if their BMI was at or above 95^th^ percentile of the 2000 CDC sex-specific BMI-for-age growth charts.

c Using a Bonferonni correction for multiple testing (15 tests), we considered p-values less than 0.003 to be statistically significant.

## Discussion

In this study, we examined the relationship between PON1 genotype and status with obesity in young Mexican-American children from the Salinas Valley, CA. Increasing number of *PON1_192_* Q alleles and increasing ARYase levels were both associated with increased obesity in young children. Despite the fact that *PON1* genotypes vary widely between ethnic groups, few studies of PON1 have accounted for potential confounding by genetic ancestry. We identified an effect of genetic confounding on the relationship between BMI Z-score and *PON1_192_* genotype that was stronger in two year old children and was not present in relation to other obesity parameters. After adjusting for genetic ancestry, the association between BMI Z-score and *PON1_192_* genotype was weaker but the same trend still remained. These results suggest that PON1 may play an important role in the heritability of obesity and demonstrate that PON1 genetic studies, especially those involving admixed populations, should adjust for potential genetic confounding.

In contrast to Veiga et al.[24], who found an increased risk of obesity in *PON1_192RR_* Portuguese adults, we observed that Mexican-American children with the *PON1_192QQ_* genotype had an increased odds of obesity. This may be due to differences in the effect of *PON1_192_* genotype in childhood versus adult obesity. Alternatively, another major difference between the two studies is the allelic distributions in the ethnic groups studied. While the frequency of the Q allele is close to 0.5 in our Mexican-American cohort, it is much higher (0.7) in the Portuguese population studied by Veiga and colleagues. Additionally, the *PON1_192_* SNP has been shown to be in linkage disequilibrium(LD) with other SNPs that can affect PON1 levels (or ARYase) to varying extents in different ethnic groups. It is possible that the relationship with *PON1_192_* may be due to LD with a different polymorphic variant. Furthermore, in a previous study, we observed slightly different patterns of LD among PON1 haplotype blocks (particularly the one containing PON1_192_) in CHAMACOS Mexicans compared to Caucasians[54]. Therefore the combination of differences in age and ethnicities studied and variation of LD patterns within these groups may help to explain the conflicting results between these studies. Other studies looking at the relationship of PON1 genotype with obesity among adults have been inconsistent. Furthermore, only one other study has been reported in children. Although they focus primarily on the effect of a gene-environment interaction between pesticide exposure and *PON1_192_* genotype, their data in unexposed children indicate a small yet statistically significant association between PON1_192_ QR/RR genotype and decreased waist circumference, body fat percentage, and BMI Z-score[26], corroborating our findings.

Although PON1 status, which accounts for both PON1 quantity and enzyme activity[46], is considered more informative than looking at genotype alone, no other studies have examined the effect of PON1 status on obesity. Interestingly, while we found both *PON1_192_* genotype and ARYase were positively associated with obesity (independent of each other) in two year olds, increasing ARYase activity was protective in *PON1_192QQ_* but not *PON1_192RR_* five year olds. One study of Hungarian children (mean age  = 12 years) also showed lower ARYase in obese children but did not report *PON1* genotype frequencies. However, if we consider results of other studies that generally observe a much higher frequency of QQ (51%) than RR(8%) genotypes in Hungarian individuals[62], our findings in five year old *PON1_192QQ_* CHAMACOS children are consistent with the study of Hungarian children, the majority of whom were likely *PON1_192QQ_.* The association between increased ARYase and obesity parameters in two year old children was unexpected. However, this relationship may have been driven by the weak but significant LD between *PON1_192_* and promoter SNPs *PON1_−108_* and *PON1_−909_*, both of which are associated with ARYase activity. Indeed, we found a modest but statistically significant relationship between ARYase and *PON1_192_* with lower ARYase activity in children with *PON1_192_* QR and RR genotypes. Among five year old children, we found a significant interaction between *PON1_192_* and ARYase activity such that higher ARYase activities in QQ children seemed to be protective against obesity. Given that QQ children are at increased risk of obesity compared to QR and RR children, this relationship indicates that children with both low ARYase and the QQ genotype may have an even higher risk of obesity than other children. Overall, these data suggest that PON1 genotype and protein expression may play a role in obesity. The biological link between the two is likely the oxidative stress pathway as it is well established that PON1 is involved in lipid peroxidation[18], [63], [64]and obesity is characterized by chronic oxidative stress[19].

To our knowledge, this is the first study of genetic associations of PON1 with obesity that has accounted for potential confounding by genetic ancestry. Since PON1 allele frequencies are quite different by ethnic groups and prevalence of obesity clearly varies between ethnic and racial groups as well, it is important to consider this critical factor. Our data demonstrate some suggestive evidence of genetic confounding by population stratification in models examining the association of PON1 with BMI Z-scores. Only one other study has used AIMs to examine the effects of genetic ancestry on PON1 [32]. However in that study, Lee and colleagues looked at a much smaller set of 35 AIMs and found adjustment for genetic ancestry provided only limited improvement in the fit of the models of PON1 genotype with ARYase in African-American and Caucasian mothers and their children. Accounting for the contribution of genetic ancestry may be more critical when considering health outcomes that vary broadly between ethnic groups such as birth weight, obesity, and cardiovascular disease.

Interestingly, the associations observed at ages 2 and 5 were quite different. For instance the relationship between *PON1_192_* genotype and obesity was stronger and the effect of genetic confounding was more noticeable at age 2 compared to age 5. This data however corroborates well with data from twin and adoption studies which indicate that the heritability of obesity is lowest at age 5 when the effect of common environmental factors is strongest[65]. Along these lines, one would expect that the association of obesity with both genetic factors (e.g. PON1 genotype and genetic ancestry) would be more prominent at age 2 compared to age 5, as we observed in our cohort.

As a secondary analysis, we also examined the direct association between genetic ancestry and obesity parameters. We found that increased African ancestry was marginally associated with higher BMI Z-scores and odds of obesity. These results are similar to those reported by Fernandez et al.[33] and Tang et al. [34], which showed positive associations between African ancestry estimates and obesity parameters in adults. These data provide further evidence that it is important to adjust for ancestry in genetic studies of obesity.

Although we identified some meaningful relationships of obesity with both PON1 and genetic ancestry, this study does have some limitations. First, it is well established that obesity is multifactorial in nature, so while we found that genetics explains some of the variance in obesity parameters, other factors such as environmental obesogens, gene-environment interactions, and epigenetics should be considered in the future. Also, here we focused exclusively on one candidate gene, *PON1*, and it may be useful to explore other related genes, including those involved in lipid peroxidation and oxidative stress pathways. Despite these limitations, our findings suggest an intriguing role of PON1 in obesity and underscore the importance of accounting for differences in genetic ancestry in studies of PON1 and health outcomes.
